# The Influence of Strain Aging at Different Temperatures on the Mechanical Properties of Cold-Drawn 10B21 Steel Combined with an Electron Microscope Study of the Structures

**DOI:** 10.3390/ma17040826

**Published:** 2024-02-08

**Authors:** Qiuyao Dong, Hengchang Lu, Yangxin Wang, Xianliang Yang, Linxiang Zhang, Han Dong

**Affiliations:** 1 School of Materials and Science Engineering, Shanghai University, Shanghai 200444, China; dqy13479014616@163.com (Q.D.); flypenguin@shu.edu.cn (Y.W.); donghan@shu.edu.cn (H.D.); 2 Zhejiang Institute of Advanced Materials, Shanghai University, Jiaxing 314100, China; yangxianliang001@163.com; 3 Technology Centre of Jiangsu Yonggang Group Company Limited, Suzhou 215600, China; zhangxindeailian@163.com

**Keywords:** aging treatment, cold drawn, dislocation, strength, microstructure

## Abstract

The effect of aging treatments at various temperatures on the mechanical properties and microstructure of 10B21 cold heading steel with a 20% reduction in area (ε = 0.1) was investigated. The mechanical properties were evaluated based on tensile tests and hardness tests, while the evolution of microstructure was observed by using an optical microscope (OM), scanning electron microscope (SEM), transmission electron microscope (TEM) and X-ray diffraction (XRD). The results reveal that aging treatment enhance the strength and hardness of 10B21 cold heading steel after drawing, and the highest values of strength and hardness are attained at an aging temperature of 300 °C. Specifically, the yield and ultrahigh tensile strength after aging at 300 °C are measured at 620 MPa and 685 MPa, respectively, which are 30 MPa and 50 MPa higher than the cold-drawn sample. Moreover, the hardness after aging at 300 °C reaches 293 HV, which has an increase of 30 HV compared to the cold-drawn state. The improvement in mechanical properties may be related to the strain-aging mechanism and the increased density of dislocations. In addition, the analysis of the TEM results reveal that the presence of the second-phase Ti(C,N) contributes to pinning the dislocations, whereas the dislocations are pinned between the cementite (Fe_3_C) lamellar and stacked at the grain boundaries, leading to strain hardening of the material.

## 1. Introduction

Non-quenched and tempered steels are alloyed by low or medium carbon with microelements (V, Ti, Nb, etc.), followed by controlled temperature forging and cooling to disperse carbide- or nitride-strengthening phases from ferrite and pearlite, without quenching and tempering treatment [1,2,3]. 10B21 steel is the grade in the American standard ASTM A510/510M [4], which is currently on the market for 8.8-grade fasteners. In general, when using 10B21 steel to process fasteners, it is usually spheroidized and annealed or softened before drawing to reduce strength, increase plasticity and improve its cold working properties [5]. However, the annealing process is energy-consuming and time-consuming, which not only increases the cost but also pollutes the environment. In this regard, the market has developed a cold heading steel 10B21 coil with excellent cold working performance, which is designed and optimized from the chemical composition and process control to ensure that the hot-rolled coil obtains lower strength and higher plasticity. It has favorable cold drawing and cold heading performance, which can be exempted from the annealing process and directly drawn and cold heading formed. Cold-heading steel typically comprises low- to medium-carbon high-quality carbon structural steel and high-quality alloy structural steel. It is used for cold heading forming to manufacture various mechanical standard components and fasteners. This type of steel is employed in the production of high-standard, non-standard and special-shaped components using the cold heading forming process under room-temperature conditions. The primary application of cold heading steel is in the production of various mechanical foundational components, primarily used for connection and fastening purposes. This includes, but is not limited to, bolts, screws, nuts, washers, pins, self-tapping screws, rivets and assembled components.

However, with rapid development in recent years in industries, such as automotive, machinery, construction and light manufacturing, there is an urgent need for cold heading steel with better comprehensive performance. The stability and purity of domestically produced cold heading steel in China are relatively low, leading to a significant dependence on imports, especially for cold heading steel used in the manufacturing of high-strength bolts for automobiles, where the strength needs to exceed class 12.9. Currently, domestic cold heading steel needs to both meet user requirements and elevate inspection standards. Moreover, considering domestic development, there is a need to intensify research and development efforts in non-tempered cold heading steel, dual-phase steel, high-strength boron steel, ultra-fine-grain steel, corrosion-resistant steel, etc., to promote the development and use of corresponding products. Additionally, the cold heading steel market will evolve towards environmental protection, fostering resource conservation and environmentally friendly production practices, achieving sustainable development. The future cold heading steel market will explore broader markets and prospects in the directions of high performance, high quality, environmental friendliness and innovation.

For these microalloyed steels, the strengthening effect of microalloy additions is produced by a combination of cold deformation and process hardening [6,7]. The cold drawing technique serves not only to manipulate wire dimensions and refine surface conditions but also to capitalize on strain hardening to enhance the steel strength, achieving the mechanical requirements for high-strength bolts. However, the cold deformation process induces the formation of high-density dislocations in steel, which remain in an unstable state and exhibit glide during loading, triggering minute yielding. This results in permanent extension, adversely impacting the load-bearing performance of bolts. Hence, post-cold deformation aging treatment is imperative for enhancing mechanical properties. To further improve strength, non-quenched and tempered steels are usually aged at low temperatures. During aging, tiny carbide or nitride particles are dispersed and precipitated, leading to further dispersion strengthening [8]. The strengthening mechanisms of the non-quenched and tempered steels include refinement strengthening, interface strengthening, precipitation strengthening, solution strengthening and dispersion strengthening [9,10,11]. In addition, Ti can effectively refine grains by forming carbonitride, which prevents grain growth at high temperatures due to its high dissolution temperature [12]. It is well known that defects, such as grain boundaries, interfaces, dislocations and vacancies, benefit from the nucleation of precipitates. Wang et al. [13] discussed that particles tend to be present in clusters if they form on subgrain boundaries and tangled dislocation lines. The high dislocation density in Fe-C and Fe-N martensite is known to be one of the main causes of its overall yield strength. Andric et al. [14] revealed the mechanistic origin of dislocation enhancement in Fe-C and Fe-N martensite by introducing a model. The model has been proposed for the size of Cottrell atmospheres and the number of interstitials trapped within these atmospheres, utilizing the interaction between the stress field of dislocations and the misfit tensor of interstitial elements.

Strain aging occurs when a metal or alloy undergoes plastic deformation or is aged after plastic deformation has occurred. Strain aging includes static strain aging (SSA) [15,16] and dynamic strain aging (DSA), depending on the state. Static strain aging is the process of aging after plastic deformation, while dynamic strain aging is the process of plastic deformation and aging at the same time. Strain aging occurs in relation to time and temperature and is a phenomenon that results in significant changes in the mechanical properties of steel after plastic deformation [17]. This phenomenon is due to the diffusion of interstitial nitrogen and carbon atoms, which locks the moving dislocations in their new positions after plastic deformation of the steel. The locking effect increases with the interstitial content of these elements and with the aging time. The extent of solute atom diffusion is intricately tied to temperature, with higher temperatures enhancing the diffusion capacity of atoms, leading to accelerated aging kinetics. Consequently, strain aging progresses slowly at room temperature. Increasing temperature promotes atom diffusion, facilitating the attainment of peak strength through rapid strain aging. Aging at room temperature is termed natural aging, while higher temperature aging is referred to as artificial aging. Artificial aging proceeds faster and is frequently used to simulate natural aging. At low temperatures (T < 100 °C), the solubility of N atoms in ferrite is about 100-times that of C atoms, making N atoms the primary contributors to strain aging at low temperatures. However, at higher temperatures (T > 100 °C), even after complete removal of N atoms from the solid solution, strain aging can occur due to the increased solubility of C atoms in the ferrite matrix. Further temperature increases (T > 350 °C) may induce strain aging by other solute atoms, such as P, Nb, Ni, Si and Ti. Loporcaro et al. [18] investigated the long-term effects of strain aging on low-carbon steel reinforcement. Mezo et al. [19] investigated the static strain aging phenomenon in heavily drawn high-carbon steel wires, and their corresponding cords were undertaken within a temperature range of 80–200 °C. Mishet’yan et al. [20] investigated the strain aging characteristics of low-alloy pipeline steels with different structural states (ferrite-pearlite, bainite), and an innovative approach was applied to assess the influence of strain rate during static loading on the yield strength of the steel in its initial condition and after strain aging.

In the current research endeavor, we meticulously delve into the impact of aging treatment conducted at different temperatures on the strength variations exhibited by 10B21 cold heading steel subjected to a 20% reduction in area. The primary objective of this inquiry is to comprehensively examine the influence of strain aging on the mechanical properties of the steel. This systematic exploration holds profound significance as it contributes significantly to advancing our knowledge and comprehension of 10B21 steel, shedding light on its inherent strengthening characteristics. The findings derived from this investigation are anticipated to offer valuable insights into optimizing the performance of 10B21 steel in diverse industrial applications.

## 2. Materials and Methods

The test materials are 10B21 hot-rolled discs with nominal diameter Φ16 mm, the microstructure is ferrite and pearlite and its chemical compositions are 0.20 wt% C, 0.06 wt% Si, 0.81 wt% Mn, 0.02 wt% P, 0.004 wt% S, 0.039 wt% Ti, 0.0018 wt% B. The hot-rolled state material with an initial diameter of 16mm undergoes acid pickling and phosphate saponification, followed by cold drawing on a drawing die, reducing the diameter to 14.31 mm, corresponding to a drawing reduction rate of 20%. Table 1 shows the mechanical property of 10B21 steel at hot-rolled state and as cold-drawn state.

Aging treatment is performed utilizing the Nabertherm Nat15/65 heat treatment tempering furnace. The tempering furnace temperatures are set successively at 200 °C, 250 °C, 300 °C and 400 °C. Upon reaching the designated temperatures, specimens are put into the furnace and subjected to a 2 h insulation period. Subsequent to this treatment, the samples underwent air cooling on refractory bricks, followed by the evaluation of mechanical properties and characterization of microstructural features.

Smooth tensile specimens are mainly used for conventional tensile experiments to test ultrahigh tensile strength (Rm), yield strength (Rp0.2), shrinkage in section (Z) and elongation (A) of the experimental material. The conventional tensile test is carried out at room temperature on MTS C45.305 universal tensile tester with a tensile speed of 0.2 mm/min and a strain rate of 0.00025 s^−1^. Specimens with a length of 200 mm and a gauge length of 70 mm are utilized. In order to accurately calculate yield, an extensometer with a standard distance of 50 mm is used during the test. Each test set involves three specimens.

According to GB/T 4340-2009 [21] Vickers hardness standard, Wilson VH1102+ Diamet Full Auto type Vickers hardness tester (Buehler, Lake Bluff, IL, USA) is used to test the hardness; the loading load is 1 kg, the loading time is 10 s, the HV1 hardness of the specimen is measured and the experimental results are the average of at least 10 data points. Before testing, the sample is first surface polished with coarse sandpaper to ensure that the upper and lower surfaces of the hardness specimen are parallel. Finally, it is polished with 1200# sandpaper, and the test surface needs to be polished.

The methods used in this work to characterize the microstructure of materials include scanning electron microscope (SEM), transmission electron microscope (TEM) and X-ray diffraction (XRD).

The metallographic specimen for SEM experiments was acquired by wire cutting a 15 mm cylindrical section along the length of the rod. The metallographic sample was prepared using standard procedures, involving mechanical grinding and successive mechanical polishing with silk cloth and a 2.5 μm abrasive spray. After thorough alcohol cleaning, corrosion was performed using a 4% nitric acid alcohol solution, and observations were made using a Sigma 300 field-emission scanning electron microscope (SEM) (ZEISS Group, Jena, Germany).

The preparation method for transmission electron microscope (TEM) samples involves cutting 0.5 mm thick slices along the length of the rod using a wire saw. These slices are mechanically ground with 240#~2000# sandpaper until reaching a thickness of 50~60 μm. Subsequently, the slices are punched into circular discs with a diameter of 3 mm using a sample punching machine. Dual-jet electropolishing is then employed on the circular discs using a dual-jet electropolishing instrument. A 6 vol.% alcoholic solution of high-purity perchloric acid is selected as the electropolishing electrolyte, and the experimental temperature is controlled to −20~−30 °C by the addition of liquid nitrogen to the electrolyte. The electropolishing is conducted in a constant current mode with a current range of 60~75 mA.

The X-ray diffraction (XRD) line profiles are measured on a cross-section along the rod axis using an X-ray diffractometer (Rigaku Ultima IV with a scintillation counter, Rigaku, Tokyo, Japan), operated at 40 kV and 40 mA using CuKα radiation, with a sampling interval of 0.01° and a scanning speed of 0.5°/min. The preparation criteria for XRD testing dictate that the specimens should not surpass a thickness of 10mm, demanding meticulous metallographic grinding and subsequent polishing for surface refinement.

## 3. Results and Discussion

### 3.1. Mechanical Properties

**Figure 1:**
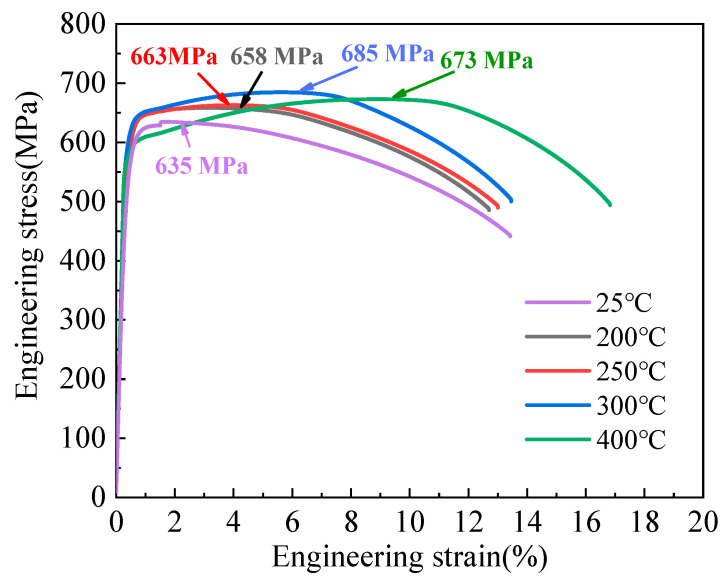
Engineering stress–strain curves.

Figure 1 shows the tensile mechanical properties of the 10B21 cold heading steel before and after aging treatment. The results show that the tensile curves exhibit continuous yielding for all different aging temperatures. In fact, tensile deformation is the process of dislocation slip; above the slip surface, the dislocation center region is compressive stress, while below the slip surface, it is tensile stress. During the tensile process, the C and N interstitial atoms interact with the dislocations and are deflected below the edge dislocations, which can offset some of the stresses, thus stabilizing the dislocations and having a “pinning effect” on the dislocation movement [22]. Upon subjecting the material to various temperature aging treatments, the interaction forces between C and N atoms and dislocations exhibit dissimilarities, influencing the hindrance levels to dislocation motion. This variability in hindrance directly affects changes in yield strength during the tensile process, thereby exerting an impact on the tensile curve.

**Figure 2:**
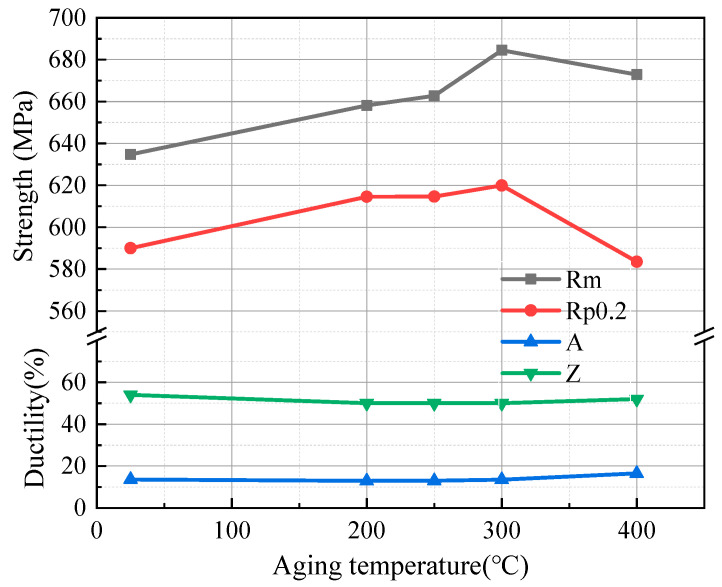
Mechanical properties of tested steel with aging temperature.

Figure 2 shows the strength and plasticity of the investigated steel before and after aging treatment at different temperatures. The results show that the yield strength (Rp0.2) and ultrahigh tensile strength (Rm) increase at first and then decrease with the increase in temperature in an aging temperature range from 200 °C to 400 °C. The yield strength and ultrahigh tensile strength are 620 MPa and 685 MPa, respectively, at an aging temperature of 300 °C, which are 30 MPa and 50 MPa higher than the cold-drawn sample. When the aging temperature reaches 400 °C, the ultrahigh tensile strength is increased by 38 MPa compared to the cold-drawn sample, but yield strength is 7 MPa lower than the cold-drawn sample. The percentage elongation (A) of the specimen in the as cold-drawn is 13.6%, and when aged at 300 °C is 13.5%, compared with the cold-drawn sample, the percentage elongation does not decrease significantly while the strength is increased, and the percentage elongation is 16.5% when the aging temperature is 400 °C, which is increased by about 3%, as compared with the cold-drawn specimen. The shrinkage in section (Z) exhibits minimal variation before and after aging treatment, indicating that the aging treatment has a limited effect on plasticity. Strain aging prompts the segregation of solute atoms within dislocation areas, contributing to an improvement in plasticity [23]. Nevertheless, as strength increases, there is a corresponding decline in plasticity. The synergy between these factors leads to a modest alteration in plasticity.

**Figure 3:**
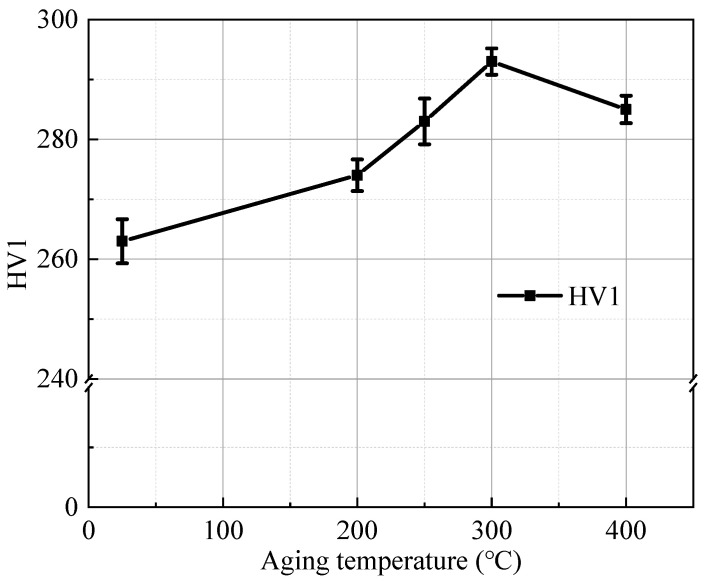
The hardness curve of tested steel with aging temperatures.

The hardness curve of the investigated steel after aging at 200–400 °C for 2 h is shown in Figure 3. Clearly, it can be seen that the hardness of the steel increases at first and then decreases as the temperature increases. The hardness peak appears at 300 °C and increases from 263 HV in the cold-drawn sample (25 °C) to 293 HV. When the temperature exceeds 400 °C, the hardness decreases gradually and reaches 285 HV, indicating that the hardness still improved compared to the hardness of the cold-drawn sample. This is due to the precipitation of carbon, nitrogen and other elements in the ferrite grains during the aging process; carbon and nitrogen atoms can diffuse to the dislocations at a shorter distance to form the Cottrell atmosphere [24], locking dislocations, thus increasing the hardness after aging treatment.

The tendency of aging is mainly due to the diffusion of C and N atoms. During the plastic deformation of the crystal, the solute atoms are affected by the high-energy strain energy generated by the distorted atomic structure around the dislocation, which can attract the solute atoms to the central region of the dislocation to form the Cottrell atmosphere. The carbon and nitrogen atoms are able to reach the dislocations and other crystal defects and stress concentrations with shorter distances, and they are enriched around the dislocations [25]. To make the dislocations continue to move, it is necessary to increase the external force, which increases the strength and hardness.

### 3.2. Microstructure

**Figure 4:**
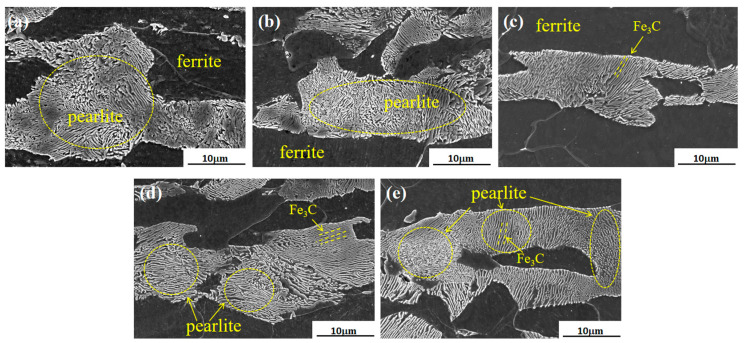
SEM images of microstructure aging at different temperatures: (**a**) cold-drawn sample, (**b**) 200 °C, (**c**) 250 °C, (**d**) 300 °C, (**e**) 400 °C.

SEM micrographs of the cold-drawn state and after different temperature aging treatments are presented in Figure 4. It is evident from the figures that the microstructure retains its composition of ferrite and pearlite following the aging treatments. In the cold-drawn state, the morphology and orientation distributions of the cementite are non-uniform. However, after the aging treatments, the lamellar structure of pearlite becomes clearer, and the orientation distribution of the cementite becomes more uniform and consistent.

**Figure 5:**
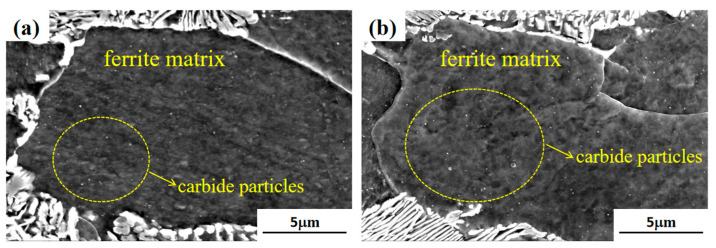
SEM images of microstructure of (**a**) cold-drawn sample, (**b**) 300 °C.

Figure 5 displays SEM images of the cold-drawn state and after 300 °C aging treatment. Analysis of Figure 5a highlights the existence of dispersed carbide particles within the ferrite matrix, which precipitated during the cold rolling process. Upon undergoing the 300 °C aging treatment shown in Figure 5b, the ferrite matrix demonstrates increased numbers and dimensions of carbide particles. This phenomenon arises from the carbon dissolution from the cementite phase to the ferrite matrix [26], resulting in the formation of small carbides.

**Figure 6:**
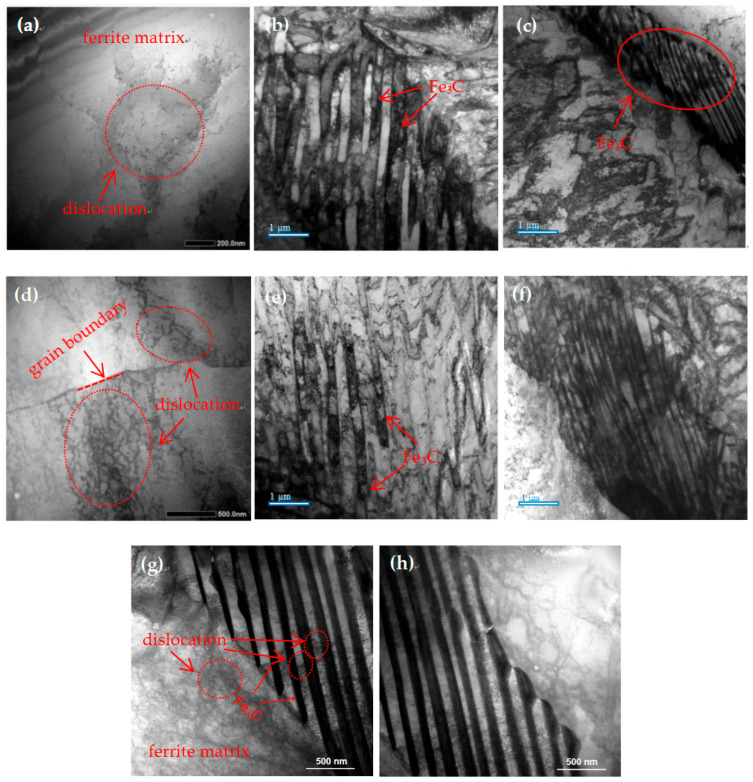
TEM images showing ferrite and lamellar cementite structure of (**a**–**c**) bright-field image of the cold-drawn specimen, (**d**–**h**) bright-field image of the specimen aged at 300 °C.

To further investigate the microstructural changes in ferrite and pearlite, TEM analysis is conducted on the experimental steel samples in the cold-drawn state and after 300 °C aging treatment. The microstructure in the cold-drawn state is depicted in Figure 6a–c, where Figure 6a illustrates the presence of randomly distributed dislocations within the ferrite matrix, which are generated during the drawing process. Figure 6b,c display the morphology of cementite within the matrix, exhibiting a uniform distribution with varying sizes. The microstructure after 300 °C aging treatment is shown in Figure 6d–g. From Figure 6a, it can be observed that grain boundaries impede the movement of dislocations, resulting in the accumulation of a considerable number of dislocations at the grain boundaries, thereby inducing strain hardening and an increase in both strength and hardness [27,28]. Figure 6e,f indicate the presence of high-density dislocations between the lamellar cementite layers. To gain a clearer understanding of the distribution of dislocations between the lamellar cementite layers, further examination at higher magnification is performed, as illustrated in Figure 6g,h. It can be seen that the dislocation density in the ferrite is extremely high, which exhibits inhomogeneous distribution characteristics, entangled into a cellular structure, and the dislocations in the ferrite are pinned by the cementite on both sides. The thin cementite lamellar is hardly broken in the rolling friction to pile up a large number of dislocations in the ferrite matrix, and the work hardening degree is improved [29,30].

**Figure 7:**
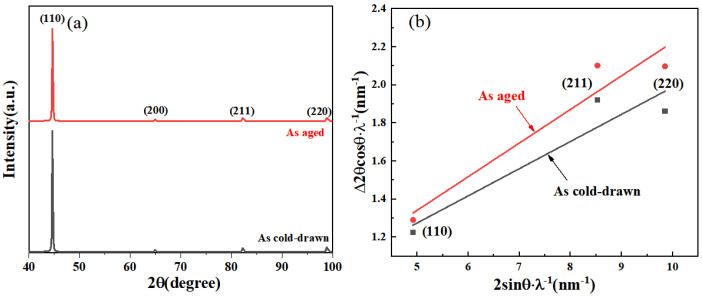
(**a**) X-ray diffraction pattern of the cold-drawn 10B21 steel aged at 300 °C. (**b**) The calculation of Williamson–Hall plots.

The XRD results of the sample before and after aging treatment are shown in Table 2 and Figure 7. In order to calculate the change in dislocation density before and after aging of the specimens, the dislocation densities were evaluated using a Williamson–Hall [31,32] plot based on the full width at half maximum (FWHM)(Δ2θ) of the reflection peaks for (110), (200), (211) and (220), and the formula for dislocation density is calculated as follows.

(1) $$\frac{∆2\theta \cos\theta }{\lambda }=\frac{0.9}{D}+2\varepsilon \frac{\sin\theta }{\lambda }$$

where *λ* is the X-ray wavelength (=0.154 nm), *D* is an apparent crystallite size and ε is a lattice strain. The calculation of Williamson–Hall plots is shown in Figure 7b.

(2) $$\rho =\frac{2\sqrt{3}\varepsilon }{Db}$$

where *b* is the magnitude of the Burgers vector, which is 0.249 nm for bcc. All the values of FWHM, 2θ, ε, *D* and *ρ* are listed in Table 2.

Table 2 illustrates the essential parameters necessary for dislocation density calculation and presents the resulting dislocation density values. Notably, the dislocation density undergoes an increase from 6.0 × 10^15^ cm^−2^ to 8.2 × 10^15^ cm^−2^ following the 300 °C aging process.

**Figure 8:**
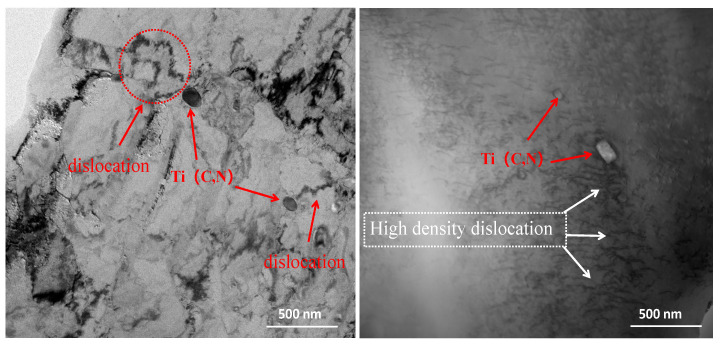
TEM images of second-phase particle distribution of the steel after aging treatment at 300 °C.

Figure 8 shows TEM images for the steel after being aged at 300 °C. It is observed that some oval or rectangle particles are precipitated in the matrix, ranging from tens to hundreds of nanometers. The carbon-nitride precipitation temperature of Ti is around 650 °C [33]. These particles are Ti(C,N), which precipitated at high temperature in the hot-rolled state [34,35], and when the particles of the second phase are uniformly distributed in the matrix phase and finely dispersed, the material undergoes a significant strengthening effect, which is called second-phase strengthening. Additionally, there are many high-density dislocations near Ti(C,N) particles, which show that the dislocation patterns of the 10B21 steel after being aged at 300 °C correspond to dislocation entanglement and dislocation pinning. Dislocations interact and prevent further movement of dislocations, thereby improving the strength of the steel. Many dislocations proliferate inside the matrix, and the proliferating dislocations move in the matrix, forming clumps with other dislocations in the matrix. According to the law of the pinning effect [36], precipitates can also pin dislocation recombination and grain boundary motion. These high-strain-rate deformation mechanisms work together to strengthen the material [37].

Aging strengthening preserves the internal structural characteristics of the microstructure. However, during the aging treatment process, carbon and nitrogen atoms in carbonitrides and solute carbon within the ferrite matrix can diffuse to dislocations at shorter distances. This results in the formation of the Cottrell atmosphere, effectively pinning dislocations and impeding their motion. All this occurs on the basis of the original characteristics, resulting in increased strength and hardness. In terms of the interaction between the dislocated phase and the strain field of the substrate co-grid, the dislocation motion generates the anti-phase domain boundary [38], so that the dislocation cannot pass through the dislocated phase; bending around to form the dislocation ring can also produce reinforcement.

## 4. Conclusions

This study investigates the effect of aging treatment at different temperatures on the strength variation of 10B21 cold heading steel with a 20% reduction in area, aiming to provide insights into the strengthening characteristics and mechanical properties of 10B21 steel. The findings are as follows:

(1) After being aged at 300 °C, the yield strength and ultrahigh tensile strength are 620 MPa and 685 MPa, respectively, which are 30 MPa and 50 MPa higher than the cold-drawn sample; the hardness after aging at 300 °C reaches 293 HV, with an increase of 30 HV compared to the cold-drawn state. There is little variation in both elongation and shrinkage in the section before and after the aging process, so the impact of aging treatment on plasticity is minimal.
(2) Cold deformation generates dislocations whose density after aging treatment increases from 6.0 × 10^15^ cm^−2^ to 8.2 × 10^15^ cm^−2^. Some of these dislocations that are pinned between reticulated carburite restrict the breaking of cementite lamellar, generating a pile up of dislocations in the ferrite matrix. Some of them are hindered by grain boundaries, accumulating at grain boundaries and causing strain hardening in the material.
(3) The improvement in tensile strength and yield strength by aging treatment at 300 °C is explained by the Cottrell atmosphere impeding dislocation motion in the strain-aging mechanism and the increased density of dislocations. Additionally, the presence of the second-phase Ti (C,N) plays the role of pinning dislocations, which leads to an increase in strength and hardness.

## Figures and Tables

**Table 1:** Mechanical property of hot-rolled state and as cold-drawn state.

State	Rm [MPa]	Rp0.2 [MPa]	A [%]	Z [%]
Hot-rolled	497	257	35.5	63
Area reduction of 20%	635	590	13.5	54

**Table 2:** The main parameters for calculating dislocation density.

Parameters	Crystal	FWHM	2θ	D	ε	*ρ*
300 °C 2 h	(110)	0.215	44.64	450	0.17673	8.2 × 10^15^
	(211)	0.43	82.241	252		
	(200)	0.479	98.84	251		
Unaged	(110)	0.204	44.621	484	0.14278	6.0 × 10^15^
	(211)	0.393	82.22	277		
	(200)	0.441	98.82	285		

## Data Availability

Data are contained within the article.
